# Diarrhea-Associated Intestinal Microbiota in Captive Sichuan Golden Snub-Nosed Monkeys (*Rhinopithecus roxellana*)

**DOI:** 10.1264/jsme2.ME17163

**Published:** 2018-09-29

**Authors:** Hui Zhu, Dong Zeng, Qiang Wang, Ning Wang, Bo Zeng, Lili Niu, Xueqin Ni

**Affiliations:** 1 Animal Microecology Institute, College of Veterinary, Sichuan Agricultural University Chengdu, Sichuan China; 2 Sichuan University of Science and Engineering Zigong, Sichuan China; 3 Chengdu Wildlife Institute, Chengdu Zoo Chengdu, Sichuan China; 4 Department of Parasitology, College of Veterinary, Sichuan Agricultural University Chengdu, Sichuan China; 5 Farm Animal Genetic Resources Exploration and Innovation Key Laboratory of Sichuan Province, Sichuan Agricultural University Chengdu, Sichuan China

**Keywords:** Sichuan golden snub-nosed monkey, intestinal microbiota, illumina MiSeq sequencing, diarrhea

## Abstract

Diarrhea is often associated with marked alterations in the intestinal microbiota, termed dysbiosis; however, limited information is currently available on the intestinal microbiota in captive golden snub-nosed monkeys (*Rhinopithecus roxellana*) with diarrhea. We herein characterized the fecal microbiota in diarrhea and healthy monkeys using the Illumina MiSeq platform. The concentrations of fecal short-chain fatty acids (SCFAs) and copy numbers of virulence factor genes were also assessed using gas chromatography and quantitative PCR (qPCR), respectively. The results obtained showed that diarrhea monkeys harbored a distinctive microbiota from that of healthy monkeys and had 45% fewer *Bacteroidetes*. Among healthy subjects, old monkeys had the lowest relative abundance of *Bacteroidetes*. Linear discriminant analysis coupled with the effect size (LEfSe) and canonical correlation analysis (CCA) identified significant differences in microbial taxa between diarrhea and healthy monkeys. A PICRUSt analysis revealed that several pathogenic genes were enriched in diarrhea monkeys, while glycan metabolism genes were overrepresented in healthy monkeys. A positive correlation was observed between the abundance of nutrition metabolism-related genes and the individual digestive capacities of healthy monkeys. Consequently, the abundance of genes encoding heat stable enterotoxin was significantly higher in diarrhea monkeys than in healthy monkeys (*P*<0.05). In healthy subjects, adult monkeys had significant higher concentrations of butyrate and total SCFAs than old monkeys (*P*<0.05). In conclusion, the present study demonstrated that diarrhea had a microbial component and changes in the microbial structure were accompanied by altered systemic metabolic states. These results suggest that pathogens and malabsorption are the two main causes of diarrhea, which are closely related to the microbial structure and functions.

The human/animal gastrointestinal (GI) tract is populated by a complex community of microorganisms that plays a pivotal role in maintaining host health and well-being (10, 49). The intestinal microbiota is crucially involved in extracting nutrients from the diet, which influences host nutrition, metabolism, and body development (50). It may also prevent the colonization of pathogens in the GI tract and is essential for mucosal homeostasis, intestinal maturation, and full functionality (9). Previous studies indicated that a number of GI diseases, including those involving diarrhea, are often associated with specific alterations in microbiota components, called dysbiosis (41). GI diseases are considered to be driven, at least in part, by alterations in the microbiota; however, it currently remains unclear whether dysbiosis is the cause or a consequence of these diseases (11, 33).

The golden snub-nosed monkey (*Rhinopithecus roxellana*) is a rare and endangered primate under first-class national protection in China. Its distribution is restricted to temperate montane forests at 1,400–3,000 m above sea level across three isolated regions in central and southwestern China (24). Due to their ecological status and research value as a close reference for human health, studies on the health of golden snub-nosed monkeys have attracted worldwide attention. The golden snub-nosed monkey is an Old World monkey in the Colobinae subfamily, which have significantly longer gastrointestinal tracts and larger stomach surface areas than other mammals (20). Wild golden snub-nosed monkeys eat fiber-enriched plants as their staple food (19). However, the diet of captive golden snub-nosed monkeys cannot completely mimic the natural diet of wild moneys, and may contain less fiber or nutrients. Changes in living conditions, particularly in diet, may negatively affect the growth and well-being of golden snub-nosed monkeys, including the induction of gastrointestinal diseases. Gastrointestinal diseases, such as diarrhea, enteritis, and constipation, are the most frequently occurring diseases (60–80%) among captive monkeys, and according to long-term clinical records in Chengdu Zoo, diarrhea is more common in old monkeys. In daily feeding, most captive monkeys have the status of sub-health for long-term or accidental diarrhea, accompanied by decreases in digestion and metabolism, the loss of appetite, and growth retardation. Even though sub-health has not threatened the survival or breeding of golden snub-nosed monkeys, it endangers the health and welfare of this rare species. In order to improve the health of captive golden snub-nosed monkeys, a healthy microbiota in the GI tracts of these monkeys needs to be established in order to prevent diarrhea. Therefore, the present study was conducted in order to characterize the intestinal microbiota of Sichuan golden snub-nosed monkeys in relation their ages and health status. We hypothesize that: i) the components and structures of the intestinal microbiota of healthy and diarrhea monkeys differ, ii) old monkeys have a microbial component that is susceptible to diarrhea, and iii) pathogens and malabsorption are the two main causes of diarrhea and are closely related to the microbial structure and functions.

## Materials and Methods

### Experimental design and sampling

Our experiment was conducted in the Chengdu Zoo (Chengdu, Sichuan, China), which harbored a captive population of 18 golden snub-nosed monkeys. Their daily diet consisted of approximately 500–800 g of green fodder (privet leaves, mulberry, fruit branches, perennial ryegrass, and Sudangrass), 300–500 g of seasonal fruits and vegetables, and 150–200 g of concentrate (including corn, fish meal, wheat middling, soybean meal, milk, and trace mineral elements). During the experimental period of 18 months, all golden snub-nosed monkeys were monitored with a focus on the occurrence of diarrhea. Sampling work was accomplished by specific zoo keepers in Chengdu for the purpose of animal conservation. All diarrhea samples were collected and its incidence was recorded. Diarrhea was defined as loose or liquid stools, with stools classified as 5, 6, or 7 on the Bristol stool chart (3). Large numbers of samples from healthy animals (from different subjects on the same sampling day or recovered subjects) were also collected. The average life expectancy of golden snub-nosed monkeys in Chengdu Zoo is 12 years and they reach sexual maturity at approximately 3–4 years. Thus, monkeys aged 1–3 years were defined as young monkeys and those aged 4–6 years were adult monkeys. Monkeys older than 6 years and accompanied by decreases in physiological function were defined as old monkeys. Detailed information on sequenced samples was shown in Table S1. Fresh fecal samples were immediately collected upon defecation around feeding time, sealed in sterile plastic bags, transported to the laboratory in liquid nitrogen, and stored at −70°C until analyzed.

### Ethics statement

All animals were handled in strict accordance with the animal protection law of the People’s Republic of China (a draft animal protection law was released on September 18, 2009). All procedures were performed in accordance with the rules for the Care and Use of Laboratory Animals of the Animal Ethics Committee of Sichuan Agricultural University (Ya’an, China) (Approval No. 2013-028). All methods were performed in accordance with relevant guidelines and regulations, including any related details.

### DNA extraction and sequencing

All samples subjected to DNA extraction were obtained from inside feces using sterile tweezers to avoid soil contamination and had an equal weight of 75 mg. Total genomic DNA was extracted from fecal samples using the E.Z.N.A^®^ Stool DNA Kit (Omega Biotechnology, Norcross, GA, USA) according to manufacturer’s instructions, and stored at −70°C for further analyses. The sequencing of partial 16S RNA genes was performed by Novogene Bioinformatics Technology (Beijing, China). Briefly, DNA was amplified using the 515f/806r primer set (515f: 5′-GTG CCAGCMGCCGCGGTA A-3′, 806r: 5′-XXX XXXGGACTACHV GGGTWT CTA AT-3′), which targeted the V4 region of microbial 16S rRNA, with the reverse primer containing a 6-bp error-correcting barcode unique to each sample. A PCR reaction was performed using the Phusion high-fidelity PCR Master mix (New England Biolabs, Beijing, China) under the following conditions: 94°C for 3 min (1 cycle), 94°C for 45 s/50°C for 60 s/72°C for 90 s (35 cycles), and a last step of 72°C for 10 min. PCR products were purified using the QIAquick Gel Extraction Kit (QIAGEN, Dusseldorf, Germany). Sequencing was conducted on an Illumina MiSeq 2×250 platform according to protocols described by Kozich *et al.* (25).

### Bioinformatics and statistical analysis

Sample reads were assembled by the Mothur software package (44), and then quality filtered and demultiplexed in QIIME using default settings as described by Bokulich *et al.* (4). Chimeric sequences were removed by UCHIME (14). Operational Taxonomic Units (OTUs) were selected using the *de novo* OTU picking protocol with a 97% similarity threshold in QIIME software. The taxonomy assignment of OTUs was performed by comparing sequences to the Greengenes database (gg_13_5_otus). A bipartite network was used to display and analyze how OTUs partitioned between samples. Files and statistics of the network were generated by script “make_ otu_network.py” in QIIME. In order to cluster OTUs and samples in the network, a stochastic spring-embedded algorithm was used, as implemented in Cytoscape 3.2.0 (46).

An alpha diversity analysis included the Shannon index, Chao1, and Observed species. Jackknifed beta diversity included unweighted and weighted Unifrac distances calculated with 10 subsampling levels, and these distances were visualized by a Principal Coordinate Analysis (PCoA) (32). The Mann-Whitney U test and type KW test were used as significance tests of alpha diversity. A permutational multivariate analysis of variance (PERMANOVA) and analysis of similarity (ANOSIM) were used as significance tests of beta diversity differences between sample groups. A linear discriminant analysis coupled with the effect size (LEfSe) and canonical correlation analysis (CCA) (5) were performed in order to identify microbial taxa differentially represented between groups at the genus or OTU level (45). The functional profiles of microbial communities were predicted using PICRUSt (27). The bootstrap Mann-Whitney U test and type KW test were also used to identify gene pathways with significantly different abundance between groups. The R packages “Phyloseq”, “biom”, and “pheatmap” were used for data analysis and plotting (37).

### Quantitative PCR of virulence factor genes

Virulence factor genes were analyzed using the primers listed in Table S2. qPCR was performed on a Bio-Rad CFX96™ real-time PCR Detection System with CFX Manager Software version 2.0 (Bio-Rad Laboratories, Hercules, CA, USA). Each reaction was run in duplicate in a volume of 25 μL in low 96-well white PCR plates sealed with optical flat 8-cap strips (Bio-Rad Laboratories).

The SybrGreen-based reaction mixture consisted of 12.5 μL SYBR^®^ Premix Ex Taq™ II (TaKaRa Biotechnology, China), 1 μL of each primer (20 mM, Invitrogen Life Technologies, China), and 1 μL of template DNA of fecal samples (the non-template control used water). The amplification program included an initial denaturation at 95°C for 5 min followed by 40 cycles of denaturation at 95°C for 15 s, primer annealing at the optimal temperatures for 30 s and primer extension at 72°C for 30 s, 1 cycle of 95°C for 1 min, 1 cycle at 55°C for 1 min, and a stepwise increase in temperature from 55 to 95°C (at 10 s per 0.5°C) in order to obtain melt curve data. Data were collected at the extension step. Melting curves were checked after amplification and the sizes of PCR products were verified using agarose gels in order to ensure correct amplification results. Standard curves were generated from PCR amplicons according to Metzler-Zebeli *et al.* (38).

The master mixes of PCR reactions using Taqman probes contained 12.5 μL of Premix Ex Taq (TaKaRa Biotechnology), 2 μL of template DNA, 0.5 μL of each primer, and 1 μL of the probe. The PCR cycle was set to 50°C for 2 min and 95°C for 10 min, followed by 45 cycles at 95°C for 15 s and 60°C for 1 min.

### Fecal SCFA analysis

Fecal SCFA concentrations (acetate, propionate, and butyrate) were assessed by gas chromatography as described previously (48).

## Results

### Metadata and sequencing

Fifty-two fecal samples were collected from diarrhea (*n*=15) and healthy (*n*=37) golden snub-nosed monkeys, and heathy subjects consisted of young (*n*=12), adult (*n*=12), and old (*n*=13) monkeys. After removing low quality reads and chimeras, 930,794 high quality reads remained with an average of 17,899.885±7,323.639 tags per sample, ranging between 8,264 and 49,798. These sequences, with an average length of 252 bp, were assigned to 26,182 OTUs based on 97% similarity. In the downstream alpha and beta diversity analyses, the sequence number was normalized to 8,530 by randomly subsampling to standardize sampling efforts. The subsampling of sequences still yielded the sufficient resolution of microbial communities, as suggested by average Good’s coverage, ranging between 91.4±1.1% and 93.2±0.5% (mean±SD).

**Fig. 1:**
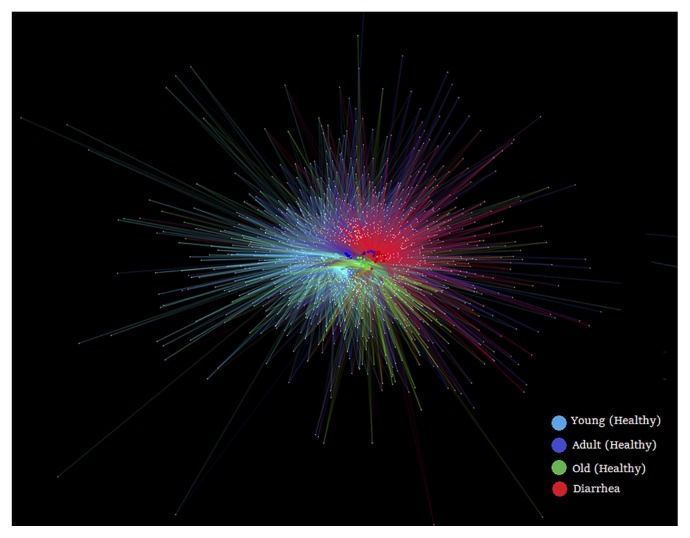
Bipartite network analysis of bacterial communities from diarrhea and healthy golden snub-nosed monkeys of different ages. OTUs and samples were designated as nodes, in which external white small nodes represented OTUs and internal colored nodes represented samples. Host node colors highlight monkey sample types. The OTU node was connected to the sample when its sequence was found in the sample.

In order to obtain an overview of bacterial genera partitioning across the host, we constructed a bipartite network (Fig. 1). The visual output of this analysis was a clustering of samples according to their shared OTUs, such that samples that share more OTUs clustered closer together. For example, old monkeys (green nodes) were closer to monkeys with diarrhea (red nodes) than other monkeys. This result was consistent with the database of shared OTUs (Fig. S1), which showed that 60.55% OTUs of old monkeys, 57.6% OTUs of adult monkeys, and 50.5% OTUs of young monkeys were shared with monkeys with diarrhea.

### Differences in microbial communities between diarrhea and healthy golden snub-nosed monkeys

**Fig. 2:**
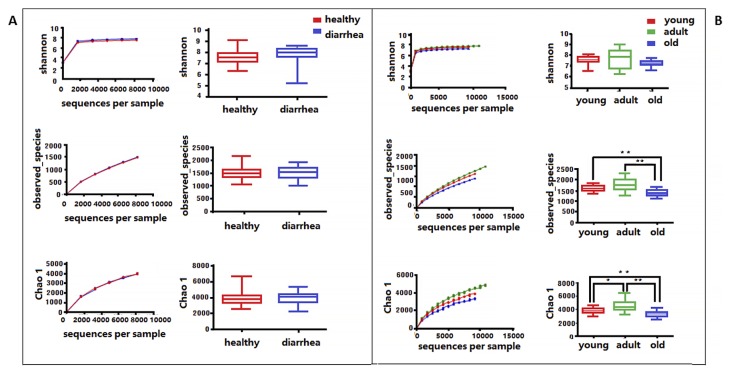
Comparison of bacterial community diversity and richness between diarrhea and healthy golden snub-nosed monkeys of different ages. Diversity and richness were measured by the Shannon index, Observed species, and Chao 1, respectively. (A): Community diversity and richness between diarrhea and healthy monkeys. (B): Community diversity and richness among healthy monkeys of different ages. The asterisk above the boxplots shows significant differences between groups (** *P*<0.01, * *P*<0.05, Mann-Whitney U test).

**Fig. 3:**
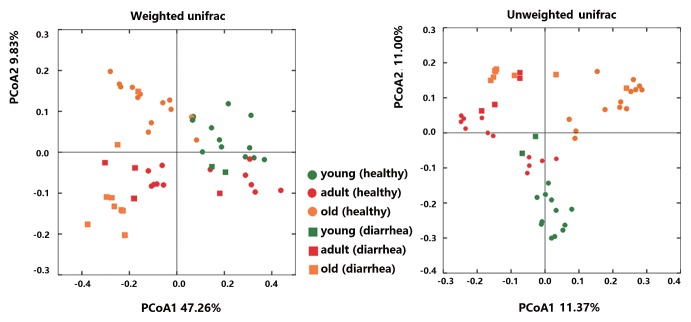
Principal Coordinate Analysis (PCoA) of weighted and unweighted Unifrac distances of 16S rRNA genes.

Microbial community richness (alpha diversity) was measured by the Shannon index, Chao1, and Observed species. No significant difference was observed between diarrhea and healthy monkeys (Fig. 2a, bootstrap Mann-Whitney test, *P*>0.05). In order to examine beta diversity measures between diarrhea and healthy monkeys, weighted and unweighted UniFrac distances were both calculated to estimate dissimilarities in the community membership. PCoA was applied to visualize distances and showed that diarrhea and healthy monkeys harbored distinct microbial taxa (Fig. 3). Based on membership, microbial communities from diarrhea monkeys clustered together and were separate from those from healthy monkeys along the principal coordinate axis. PERMANOVA (F=3.49, *P*<0.001) revealed significant differences in the community membership between diarrhea and healthy monkeys.

**Fig. 4:**
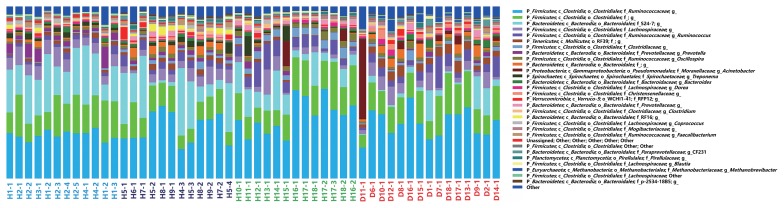
Relative abundance of OTUs at the genus level in bacterial communities from diarrhea and healthy golden snub-nosed monkeys of different ages. In the stacked bar chart, each bar represents the average relative abundance of each microbial taxon. Sample ID colors highlight the monkey sample types: young healthy monkeys marked in blue green, adult healthy monkeys marked in blue, old healthy monkeys marked in green, and diarrhea monkeys marked in red.

The community compositions of diarrhea and healthy monkeys (genus level) were shown in Fig. 4. Two groups of bacteria were dominant in the intestinal microbiota of captive Sichuan golden snub-nosed monkeys: *Bacteroidetes* and *Firmicutes*, which accounted for more than 85% of reads. The relative abundance of these two predominant microbial divisions (phylum level) in monkeys differed between healthy and diarrhea animals: monkeys with diarrhea had 42% fewer *Bacteroidetes*, and correspondingly more *Firmicutes*, than those without diarrhea.

**Fig. 5:**
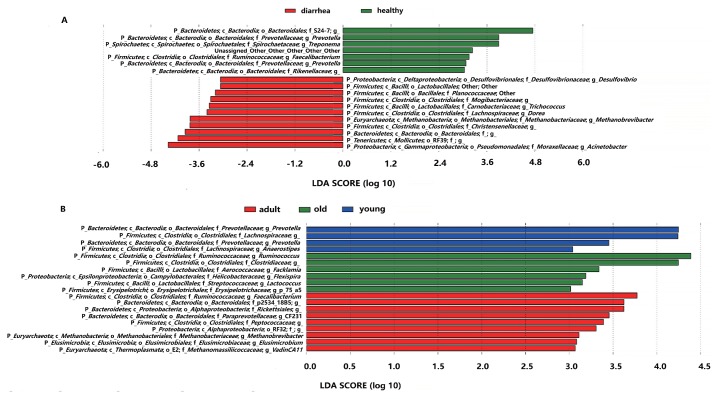
Bacterial taxa differentially represented between diarrhea and healthy golden snub-nosed monkeys of different ages identified by a linear discriminant analysis coupled with the effect size (LEfSe). (A): Different taxa (more abundant genus) between diarrhea and healthy monkeys. (B): Different taxa among healthy monkeys of different ages.

LEfSe was employed to identify specific genera that were differentially distributed between diarrhea and healthy monkeys, and is a robust tool that focuses not only on the significance of differences, but also biological relevance (45). The results of LEfSe were shown in Fig. 5a. Eighteen genera were differentially represented between the two groups, with 11 being more abundant in diarrhea monkeys (*e.g.*, *Desulfovibrio* spp., *Lactobacillales*, *Planococcaceae*, *Mogibaceriaceae*, *Trichococcus* spp., *Dorea* spp., *Methanobrevibacter* spp., *Christensenellaceae*, *Bacteroidales*, RF39, and *Acinetobacter* spp.) and 7 being more abundant in healthy monkeys (*e.g.*, S24-7., *Prevotella* spp., *Treponema* spp., *Faecalibacterium* spp., and *Rikenellaceae*). It is important to note that 6 out of 11 genera in diarrhea monkeys were members of the phylum *Firmicutes* (only 1 out of 7 genera were *Firmicutes* in healthy monkeys) and 4 out of 7 genera in healthy monkeys belonged to the phylum *Bacteroidetes* (only 1 out of 11 genera were *Bacteroidetes* in diarrhea monkeys).

In order to verify the results of the LEfSe analysis, CCA at the OTU level was conducted and the results obtained were shown in Fig. S3. The results of CCA were generally consistent with the LEfSe analysis. For example, the two OTUs (OTU48951 and 33567) close to the factor “diarrhea” belong to the order *Clostridiales* and genus *Acinetobacter*, respectively, which were significantly abundant microbial taxa in monkeys with diarrhea in the LEfSe analysis. Similarly, the two OTUs (OTU71071 and 2590) close to the factor “healthy” belonged to the family *Ruminococcaceae* and S24-7, respectively, which were also significantly abundant microbial taxa in healthy monkeys.

In order to estimate the putative role of intestinal bacteria in the health of monkeys, PICRUSt in combination with QIIME was used to predict the functional composition of a metagenome using marker gene data and a database of reference genomes, which provide direct information on a community’s functional capabilities. Metagenomic inference indicated that diarrhea monkeys harbored microbiomes with a greater abundance of disease-related genes, such as “*Staphylococcus aureus* infection”, and other genes, including “Metabolism of xenobiotics by cytochrome P450”, “Fluorobenzoate degradation”, “Drug metabolism-cytochrome P450”, “Caprolactam degradation”, “Carotenoid biosynthesis”, and “Retinol metabolism”. Healthy monkeys had more genes such as “Other glycan degradation”, “Glycosphingolipid biosynthesis-ganglio series”, and “Glycosaminoglycan degradation”, as shown in Fig. S4.

**Fig. 6:**
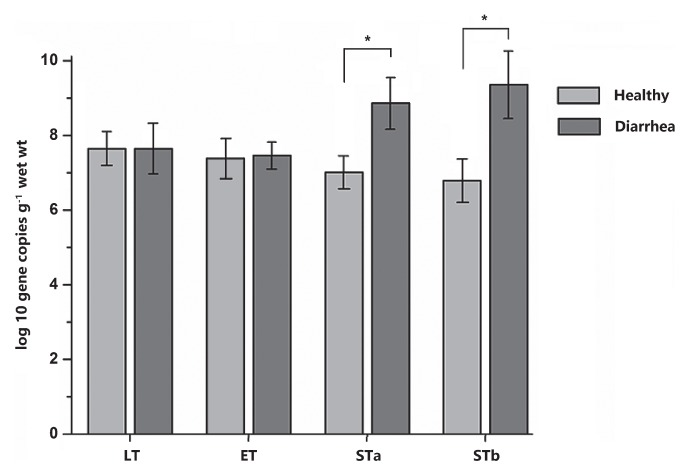
Gene copy numbers of virulence factors (heat-stable enterotoxins [STa and STb], heat-labile enterotoxin [LT] of enterotoxigenic *Escherichia coli*, and heat-stable enterotoxin [ET] of enteroaggregative *E. coli*) between diarrhea and healthy golden snub-nosed monkeys. Values are means±S.E.M. Data were analyzed by a one sample *t*-test. The asterisk above the bar chart shows significant differences between groups (* *P*<0.05), *n*=8 (diarrhea group) and *n*=16 (healthy group).

**Fig. 7:**
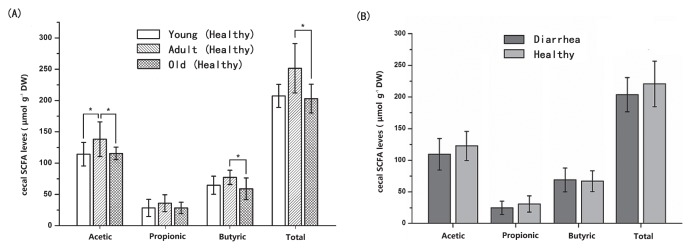
Concentrations of fecal SCFAs (acetate, propionate, and butyrate) among healthy golden snub-nosed monkeys of different ages. Values are means±S.E.M. Data were analyzed by a one-way ANOVA using the GLM procedure. The asterisk above the bar chart shows significant differences between groups (* *P*<0.05) *n*=6 (diarrhea group) and *n*=5–6/healthy age group (total *n*=16 for the healthy group).

In order to validate the results of PICRUSt, qPCR was employed to detect some common virulence factors genes. The genes encoding the heat-stable enterotoxin of enteroag-gregative *E. coli* were detected in most samples. Heat labile enterotoxin (LT) and heat stable enterotoxin EAST levels did not significantly differ between diarrhea and healthy monkey, whereas heat stable enterotoxin STa and STb levels were significantly higher in diarrhea monkeys than in healthy monkeys (*P*<0.05, Fig. 6). Staphylococcal enterotoxin A (SEA) was only identified in two samples from diarrhea monkeys and one sample from healthy monkeys (5.07, 7.10, and 5.57 log_10_ DNA gene copies g^−1^ wet weight, respectively). All samples were negative for the *Clostridium perfringens* alpha-toxin gene (*cp*A) and staphylococcal enterotoxins D (SED). In the fecal SCFA analysis, no significant differences were observed in the concentrations of acetate, propionate, and butyrate or total SCFAs between diarrhea and healthy monkeys (*P*>0.05, Fig. 7).

### Differences in bacterial communities among healthy golden snub-nosed monkeys of different ages

When alpha diversities were compared among healthy golden snub-nosed monkeys of different ages, Chao1 and Observed species indices were significantly lower in old monkeys than in young and adult monkeys (Fig. 2b, type KW test, *P*<0.01). The bacterial communities of the different age groups were clearly separated from each other by PCoA based on unweighted and weighted Unifrac (Fig. 3). Differences in community memberships among different age groups were also proven to be significant by ANOSIM (r=0.79, *P*<0.01), indicating distinct microbial community structures among monkeys of different ages. The community compositions of different ages also showed that old monkeys had the lowest relative abundance of *Bacteroidetes* within healthy subjects (Fig. 4).

The LEfSe analysis was also conducted in order to detect microbial taxa with significantly different abundance among healthy monkeys of different ages (Fig. 5b). Nineteen genera were differentially represented among the three groups (*P*<0.05), with 4 genera being more abundant in young monkeys (*e.g.*, *Prevotella* spp., *Lachnospiraceae*, and *Anaerostipes* spp.), 9 being more abundant in adult monkeys (*e.g.*, *Faecalibacterium* spp., P-2534-18B5, *Rickettsiales*, CF231 spp., *Peptococcaceae*, RF32, *Methanobrevibacter* spp., *Elusimicrobium* spp., and *vadinCA11* spp.), and 6 being more abundant in old monkeys (*e.g.*, *Ruminococcus* spp., *Clostridiaceae*, *Facklamia* spp., *Flexispira* spp., *Lactococcus* spp., and p-75-a5 spp.). Five out of six genera in old monkeys were members of the phylum *Firmicutes*. The results of the CCA analysis (Fig. S3) also support the data generated from the LEfSe analysis. For example, OTU29767 was the closest to the factor “old” and belonged to the family *Ruminococcaceae*, a significantly abundant microbial taxa in old monkeys in the LEfSe analysis.

The PICRUSt analysis was also used to investigate different metabolic potentials among healthy monkeys of different ages. By comparing their predicted metagenomes, the abundance of most nutrition metabolism-related genes appeared in the order “adult>young>old”, which indicated that adult monkeys had a stronger digestive capacity than old monkeys (Fig. S4).

Fecal SCFA concentrations were analyzed as a measure of microbial metabolic activity. Fecal concentrations of acetate were higher in adult monkeys than in young and old monkeys (*P*<0.05). Although no significant differences were observed in the concentrations of butyrate and total SCFAs between adult and young monkeys (*P*>0.05), adult monkeys had significant higher concentrations of butyrate and total SCFA than old monkeys (*P*<0.05, Fig. 7). These results were generally consistent with the results of PICRUSt.

## Discussion

In the present study, we characterized the fecal microbiota of Sichuan golden snub-nosed monkeys. The microbiota of golden snub-nosed monkeys is dominated by bacteria belonging to *Bacteroidetes* and *Firmicutes*, which account for approximately 90% of all bacteria. At the genus level, the most abundant bacteria also include some known fiber-digesting bacteria, such as *Ruminococcus*, *Prevotella*, and *Oscillospira* (13, 30). This microbiota has enabled these monkeys to adapt to flexible diets including seeds, bamboo buds, fruits, and leaves depending on the availability of food (31).

Diarrhea often causes a significant decrease in microbial diversity in humans and animals (17, 18, 47). When alpha diversities were compared between diarrhea and healthy monkeys, no significant differences were observed in diversity or richness. However, alpha diversity indices were significantly lower in old monkeys than in young and adult monkeys, which implied that the diversity of the intestinal microbiota was relevant to the stability of this micro-ecological system under normal conditions.

Distinct microbial community structures were observed between diarrhea and healthy monkeys. We simultaneously distinguished monkeys of different ages within healthy subjects. Significant differences between diarrhea and healthy subjects, as well as subjects of different ages have been observed in numerous similar studies (2, 22, 34, 39). It is important to note that diarrhea and old subjects shared a similar microbiota in the present study. In the bipartite network and 3D PCoA (Fig. S2), the distribution of nodes representing diarrhea and old subjects were closer. The significance tests of the relative abundance of OTUs, LEfSe and CCA, also revealed that diarrhea and old subjects harbored many common dominant bacteria. In CCA, the factor “old” was close to the factor “diarrhea”.

It was noteworthy that the diarrhea sample in our study was evenly collected from different age groups, although the frequency of diarrhea was the highest in old monkeys. Similarities in microbial communities between old monkeys and diarrhea samples did not appear to be attributed to diarrhea samples being collected more frequently from old monkeys. Sampling work was generally limited by the number of the population and the inhomogeneous age distribution of individuals in the zoo. Moreover, the time of defecation (the acquisition of fresh samples) was random. In the collection of diarrhea samples, multiple sampling was carefully avoided. All of the sequenced samples of diarrhea monkeys came from different individuals. According to our actual execution of the sampling scheme, once a diarrhea sample was collected, fecal samples from other healthy individuals were collected at the same time, as well as after the individual had recovered. This measure was mainly to control variations in the sampling time (seasonal factor) and individual age. Under this condition, multiple sampling was inevitable for the healthy group. However, we considered this sampling method to still be acceptable. Multiple sampling was average and random. More importantly, some of the results of the present study, as well as those of the preliminary experiment revealed by qPCR and DGGE, showed that individual difference factors had less of an influence on the intestinal microbiota than other factors including age, the sampling time, or animal physiology.

As discussed, the most obvious difference between healthy and diarrhea animals was less *Bacteroidetes* and correspondingly more *Firmicutes* in diarrhea monkeys than in healthy monkeys. The same changes were observed in old monkeys when they were compared with monkeys of other ages. Increases in the *Firmicutes*:*Bacteroidetes* ratio have also recently been reported in humans and animals with diarrhea (18, 47, 52), suggesting that dysbiosis contributed to the occurrence of diarrhea. These results demonstrated that diarrhea had a microbial component, so old monkeys with this microbial structure would be more susceptible to diarrhea.

PICRUSt predicted that the disease-related gene “*Staphylococcus aureus* infection” was overrepresented in diarrhea monkeys. We previously reported a case of a Sichuan golden snub-nosed monkey infected with *Staphylococcus aureus* in 2008 (12), but only identified SEA in two samples from diarrhea monkeys. In comparison, enteroaggregative *E. coli* appeared to be a more serious threat to animal health. The heat-stable enterotoxins of enteroaggregative *E. coli* were widespread in fecal samples and heat stable enterotoxin STa and STb levels were higher in diarrhea monkeys than in healthy monkeys.

We also found the overrepresentation of glycan metabolism-related genes in healthy monkeys than in diarrhea monkeys (Glycan is one of the most important nutritional components for Sichuan golden snub-nosed monkeys). A positive correlation was observed between the abundance of most nutrition metabolism-related genes and individual digestive capacities (young and adult monkeys had more nutrition metabolism-related genes than old monkeys, and also harbored more powerful digestive capacities). These predictions were validated by the fecal SCFA analysis, in which higher concentrations of SCFAs were observed in adult monkeys. As reported by Hale *et al.* (20), colobine monkeys, including snub-nosed monkeys, are adapted to leaf eating due to the presence of a forestomach. microbial fermentation in the forestomach plays a crucial role in their higher consumption of mature leaves (higher fiber consumption). Wild golden snub-nosed monkeys consume a number of fiber-enriched plants (large amounts of mature leaves) daily and seasonally, while captive monkeys are confronted by rapid and marked changes in diet (more fruits and concentrates), which significantly alter the gut microbiota and break the micro-ecological balance of forestomach fermentation. Thus, diarrhea in golden snub-nosed monkeys may be associated with a forestomach dysfunction. Our results support the hypothesis that pathogens and malabsorption are the two main causes of diarrhea, which are closely related to the microbial structure and functions.

These results also indicate that *Bacteroidetes* play a vital role in animal health. Previous studies reported that *Bacteroides* degrade and ferment organic matter, particularly some soluble or hydrated polysaccharides found in plant cell walls (26, 43). Members of the genus *Bacteroides* are generally considered to be the most important for pectin and lignin degradation due to their high numbers and nutritional versatility (1, 51). A comparative metagenome survey of the fecal microbiota of a breast- and plant-fed Asian elephant found unexpectedly high diversity in glycoside hydrolase family enzymes and highly abundant polysaccharide utilization loci (PULs) in *Bacteroidetes*. The baby elephant’s feces sample was dominated by bacteria belonging to *Bacteroidetes*, which accounted for more than 50% of all bacteria (the S24-7 group of *Bacteroidetes* accounted for 14%), while the relative abundance of *Bacteroidetes* (47%) decreased in six-year-old elephants (23).

Previous studies also revealed that species in the genus *Bacteroides* were involved in the glycosylation of the intestinal epithelium (7), host immune maturation (35), and protective effects against inflammation in animal models of inflammatory bowel disease and multiple sclerosis (36, 40, 42). Moreover, *Bacteroides* species were shown to be involved in species-specific physical interactions with the host that mediate stable and resilient gut colonization and some mechanisms for symbiosis (28). Decreases in *Bacteroides* have been widely reported in obesity, colorectal cancer, and enteritis or diarrhea (6, 8, 15–17, 29, 53). Therefore, the actual influence of *Bacteroides* on the physiological function of the intestines warrants further study, particularly its crucial role in host immunity and the maintenance of intestinal homeostasis.

*Lactobacillus* and *Bifidobacteria* are regarded as the most important beneficial bacteria in the GI tract (21). We also observed that the numbers of *Lactobacillus* and *Bifidobacteria* were significantly lower in diarrhea monkeys than in healthy monkeys, and were also lower in old monkeys than in monkeys of other ages by qPCR (data not shown). However, when we adopted LEfSe and CCA, due to their low biological relevance, *Lactobacillus* and *Bifidobacterium* were not differentially represented in the results of these analyses. Therefore, the relative abundance of *Bacteroidetes* may reflect the intestinal health status more intuitively, although *Bacteroidetes* still included some neutral or harmful species. As large taxa, *Bacteroides* accounted for the most substantial portion of the mammalian gastrointestinal microbiota and played a vital role in the maintenance of intestinal health. Any small change in proportion represented a large alteration in quantity, which had a marked impact on the microbial structure and intestinal ecological functions. Therefore, the relative abundance of *Bacteroidetes* (*e.g.*, the *Firmicutes*:*Bacteroidetes* ratio) has the potential to be an important biomarker for evaluating the health status of the GI.

In conclusion, the results of the present study revealed marked alterations in the intestinal microbiota of golden snub-nosed monkeys with diarrhea. Old monkeys had a similar microbial structure to diarrhea subjects. Fecal dysbiosis was associated with significant changes in the profiles of fecal SCFA and abundance of virulence factor genes, suggesting that pathogens and malabsorption are the two main causes of diarrhea.

## Supplementary Material
