# Supplementary material for: Diarrhea-Associated Intestinal Microbiota in Captive Sichuan Golden Snub-Nosed Monkeys (Rhinopithecus roxellana)
Source: Microbes Environ. 2018 Sep 29;33(3):249–56. doi: 10.1264/jsme2.ME17163 (PMC6167115; doi:10.1264/jsme2.ME17163)
Supplement: Supplementary file 1 [file 33_249_s1.pdf]

Fig. S1. Venn diagram of the unique and shared OTUs Numbers.

Fig. S2. Structures of bacterial communities were visualized by 3D Principal Coordinate Analysis (PCoA).

Fig. S3. Canonical correlation analysis of the distribution of OTUs from diarrhea and healthy golden snub-nosed monkeys in different ages.

In the CCA analysis, the distance between OTU and Factor represents its correlation. In which each small blue triangle represents an OTU (below is the OTU number), big red triangle are variable factors. The distance between OTU and factor represents its correlation.

Fig. S4. Predicted function of intestinal microbiota between diarrhea and healthy golden snub-nosed monkeys in different ages.

Values of each functional gene (row) were log2 transformed. Sample ID colors highlight the monkey sample types: young healthy monkeys marked in blue green, adult healthy monkeys marked in blue, old healthy monkeys marked in green, diarrhea monkeys marked in red. Dendrogram was generated by clustering distance row (functional gene) and column (samples) based on correlation automatically.

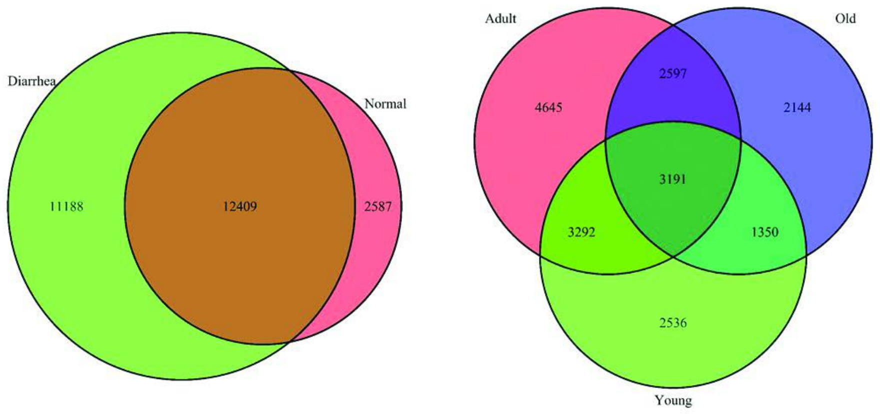
Diarrhea
Normal
11188
12409
2587
Adult
Old
4645
2597
2144
3191
3292
1350
2536
Young

Fig. S1

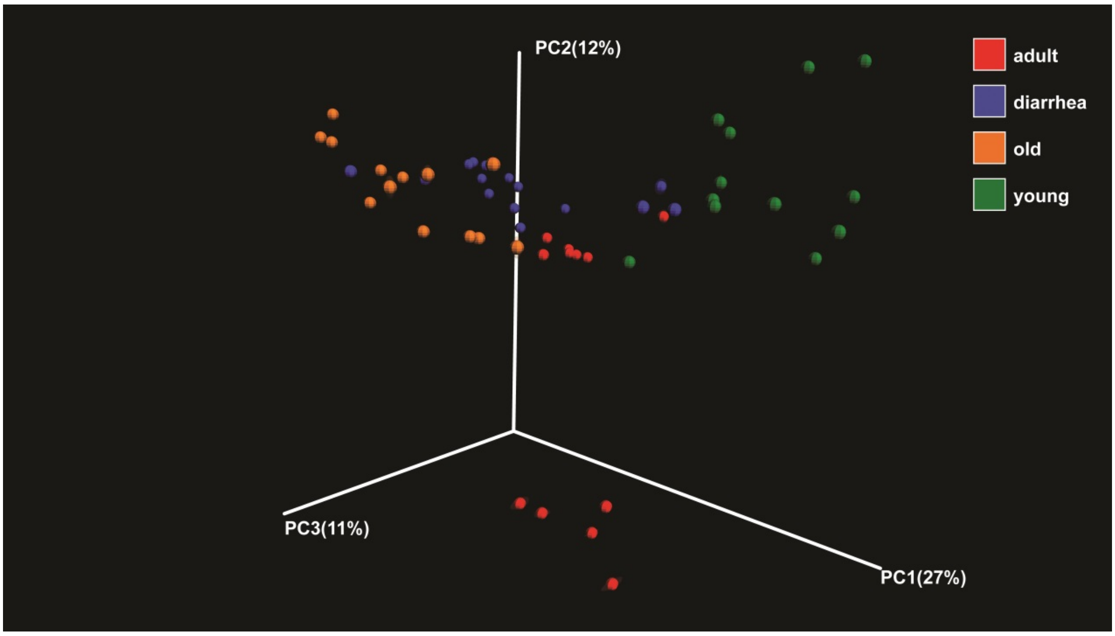

Fig. S2

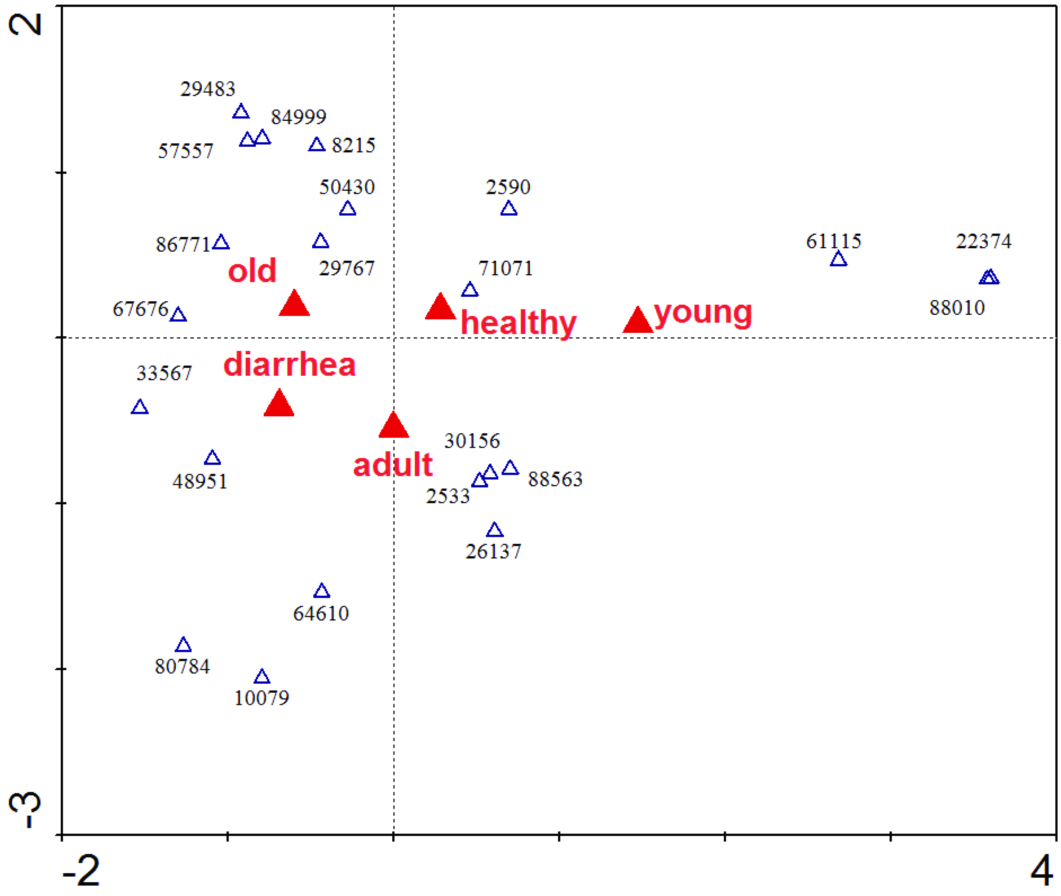

2
-3
-2
4
29483
84999
57557
8215
50430
2590
86771
29767
61115
22374
71071
old
67676
healthy
young
88010
33567
diarrhea
30156
adult
48951
88563
2533
26137
64610
80784
10079

Fig. S3

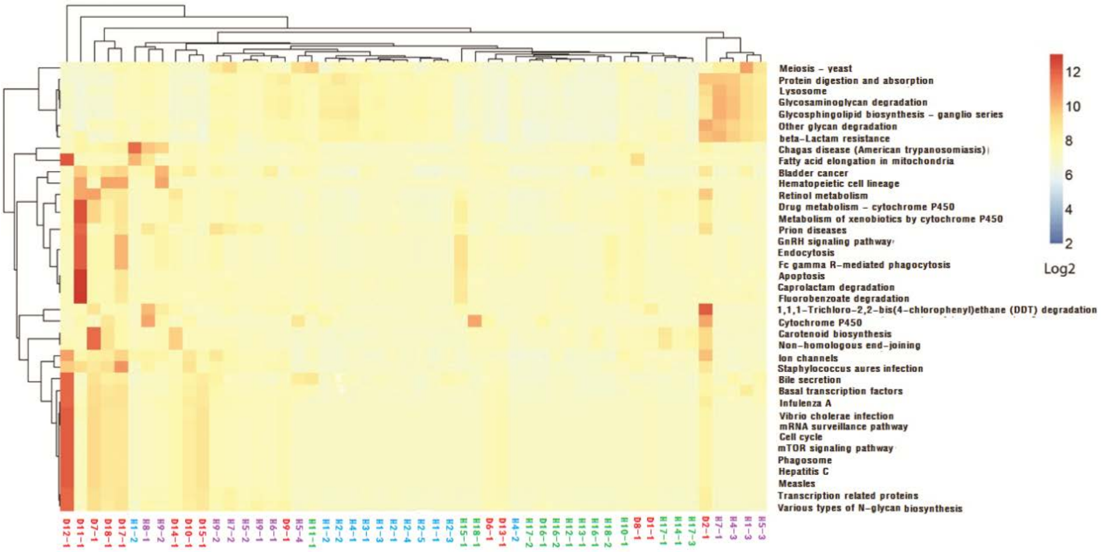
Meiosis – yeast
Protein digestion and absorption
Lysosome
Glycosaminoglycan degradation
Glycosphingolipid biosynthesis – ganglio series
Other glycan degradation
beta–Lactam resistance
Chagas disease (American trypanosomiasis)
Fatty acid elongation in mitochondria
Bladder cancer
Hematopeietic cell lineage
Retinol metabolism
Drug metabolism – cytochrome P450
Metabolism of xenobiotics by cytochrome P450
Prion diseases
GnRH signaling pathway
Endocytosis
Fc gamma R–mediated phagocytosis
Apoptosis
Caprolactam degradation
Fluorobenzoate degradation
1,1,1–Trichloro–2,2–bis(4–chlorophenyl)ethane (DDT) degradation
Cytochrome P450
Carotenoid biosynthesis
Non–homologous end–joining
Ion channels
Staphylococcus aures infection
Bile secretion
Basal transcription factors
Infulenza A
Vibrio cholerae infection
mRNA surveillance pathway
Cell cycle
mTOR signaling pathway
Phagosome
Hepatitis C
Measles
Transcription related proteins
Various types of N–glycan biosynthesis
12
10
8
6
4
2
Log2
D12–1
D11–1
D7–1
D18–1
D17–1
H1–2
H8–1
H9–2
D14–1
D10–1
D15–1
H9–2
H7–2
H5–2
H9–1
H6–1
D9–1
H5–4
H11–1
H1–2
H2–2
H4–1
H3–1
H1–3
H2–1
H2–4
H2–5
H1–1
H2–3
H15–1
H18–1
D6–1
D13–1
H4–2
H17–2
D16–1
H16–2
H12–1
H13–1
H16–1
H18–2
H10–1
D8–1
D1–1
H17–1
H14–1
H17–3
D2–1
H7–1
H4–3
H1–3
H5–3

Fig. S4

Table S1 Detailed information for sequenced samples

SampleID	IndividualName	Gender	AgeGroup	AgeDetail	SampleType	BristolScore
H1-1	huhu	female	young	2	healthy	2
H2-1	xixi	female	young	2.5	healthy	2
H2-2	xixi	female	young	2.5	healthy	3
H3-1	zaizai	male	young	2	healthy	3
H1-2	huhu	female	young	2.5	healthy	3
H2-3	xixi	female	young	2.5	healthy	3
H2-4	xixi	female	young	2.5	healthy	2
H2-5	xixi	female	young	2.5	healthy	4
H4-1	feifei	male	young	2.5	healthy	4
H4-2	feifei	male	young	3	healthy	2
H1-2	huhu	female	young	3	healthy	4
H1-3	huhu	female	young	2.5	healthy	3
H5-1	meimei	female	adult	4	healthy	3
H6-1	chengcheng	male	adult	6	healthy	3
H7-1	honghong	male	adult	6	healthy	1
H5-2	meimei	female	adult	4	healthy	2
H8-1	lele	female	adult	5	healthy	3
H9-1	tutu	female	adult	6	healthy	3
H4-3	feifei	male	adult	4	healthy	4
H5-3	meimei	female	adult	4	healthy	2
H8-2	lele	female	adult	5	healthy	3
H9-2	tutu	female	adult	6	healthy	4
H7-2	honghong	male	adult	6	healthy	1
H5-4	meimei	female	adult	4	healthy	4
H10-1	dudu	female	old	8	healthy	3
H11-1	chunchun	female	old	7	healthy	3
H12-1	qingqing	female	old	7	healthy	2
H13-1	jiajia	female	old	7	healthy	3
H14-1	yongyong	male	old	7	healthy	3
H15-1	qiqi	male	old	8	healthy	2
H16-1	longlong	male	old	9	healthy	4
H17-1	baobao	male	old	9	healthy	3
H18-1	yuyu	female	old	8	healthy	3
H17-2	baobao	male	old	9	healthy	3
H17-3	baobao	male	old	9	healthy	2
H18-2	yuyu	female	old	9	healthy	3
H16-2	longlong	male	old	10	healthy	4
D11-1	chunchun	female	old	7	diarrhea	6
D6-1	chengcheng	male	adult	6	diarrhea	6
D10-1	dudu	female	old	8	diarrhea	7

D12-1	qingqing	female	old	7	diarrhea	6
D8-1	lele	female	adult	5	diarrhea	6
D16-1	longlong	male	old	9	diarrhea	6
D15-1	qiqi	male	old	8	diarrhea	6
D1-1	huhu	female	young	2	diarrhea	7
D7-1	honghong	male	adult	6	diarrhea	6
D18-1	yuyu	female	old	8	diarrhea	6
D17-1	baobao	male	old	9	diarrhea	5
D13-1	jiajia	female	old	7	diarrhea	5
D9-1	tutu	female	adult	6	diarrhea	5
D2-1	xixi	female	young	2.5	diarrhea	5
D14-1	yongyong	male	old	7	diarrhea	5

Table S2 Primers for virulence factor genes

Target (primer size/bp)	Orientation	Primer sequence (5'-3')	Annealing temp (°C)
STa (193)	F	ATGAAAAAGCTAATGTTGGC	56
	R	TACAACAAAGTTCACAGCAG	
STb (204)	F	AATATCGCATTTCTTCTTGC	56
	R	GCATCCTTTTGCTGCAAC	
LT(291)	F	CTATTACAGAACTATGTTCGG	56
	R	TACTGATTGCCGCAATTG	
EAST1(109)	F	TGCCATCAACACAGTATATCC	56
	R	GCGAGTGACGGCTTTGT	
CPA (485)	F	GCTAATGTTACTGCCGTTGA	55
	R	CCTCATTAGTTTTGCAACC	
	Probe	FAM-GCGCAGGACATGTTAAGTTTG	
SEA(92)	F	AAAATACAGTACCTTTGGAAACGGTT	60
	R	TTTCCTGTAAATAACGTCTTGCTTGA	
	Probe	FAM-AACGAATAAGAAAAATGTAACTGTTCAGGAGTTGGATC	
SED (115)	F	TGATTCTTCTGATGGGTCTAAAGTCTC	60
	R	GAAGGTGCTCTGTGGATAATGTTTT	
	Probe	FAM-TATGATTTATTTGATGTTAAGGGTGATTTTCCCGAA	
